# Establishment of a Dual-Antigen Indirect ELISA Based on p30 and pB602L to Detect Antibodies against African Swine Fever Virus

**DOI:** 10.3390/v15091845

**Published:** 2023-08-30

**Authors:** Lei Zhou, Jinxing Song, Mengxiang Wang, Zhuoya Sun, Junru Sun, Panpan Tian, Guoqing Zhuang, Angke Zhang, Yanan Wu, Gaiping Zhang

**Affiliations:** 1 International Joint Research Center of National Animal Immunology, College of Veterinary Medicine, Henan Agricultural University, Zhengzhou 450046, China; m18294350114@163.com (L.Z.); jx-song@foxmail.com (J.S.); wmxjya@163.com (M.W.); sunzhuoya20220406@163.com (Z.S.); sunjunru152@163.com (J.S.); m13592576926@163.com (P.T.); gqzhuang2008@163.com (G.Z.); zhangangke1112@126.com (A.Z.); 2 Ministry of Education Key Laboratory for Animal Pathogens and Biosafety, College of Veterinary Medicine, Henan Agricultural University, Zhengzhou 450046, China; 3 Longhu Laboratory of Advanced Immunology, Zhengzhou 450046, China

**Keywords:** African swine fever virus, dual-antigen indirect ELISA, antibody detection, serological diagnosis

## Abstract

African swine fever (ASF) is an acute, virulent, and highly fatal infectious disease caused by the African swine fever virus (ASFV). There is no effective vaccine or diagnostic method to prevent and control this disease currently, which highlights the significance of ASF early detection. In this study, we chose an early antigen and a late-expressed antigen to co-detect the target antibody, which not only helps in early detection but also improves accuracy and sensitivity. *CP204L* and *B602L* were successfully expressed as soluble proteins in an *Escherichia coli* vector system. By optimizing various conditions, a dual-antigen indirect ELISA for ASFV antibodies was established. The assay was non-cross-reactive with antibodies against the porcine reproductive and respiratory syndrome virus, classical swine fever virus, porcine circovirus type 2, and pseudorabies virus. The maximum serum dilution for detection of ASFV-positive sera was 1:1600. The intra-batch reproducibility coefficient of variation was <5% and the inter-batch reproducibility coefficient of variation was <10%. Compared with commercial kits, the dual-antigen indirect ELISA had good detection performance. In conclusion, we established a detection method with low cost, streamlined production process, and fewer instruments. It provides a new method for the serological diagnosis of ASF.

## 1. Introduction

African swine fever (ASF) is an acute, virulent, hemorrhagic infectious disease caused by the African swine fever virus (ASFV) [1]. ASF was identified for the first time in Kenya in 1921 [2,3] and was first introduced to Europe in 1957, to the USA in 1971, and in 2007, it spread for the first time to Georgia, a country bordering Europe and Asia. The first case of ASF was confirmed in Liaoning Province, China, in August 2018, which brought huge economic losses to the pig industry [4,5]. Currently, there is no effective vaccine for ASF, and it can only be controlled in a timely manner through effective detection for diagnosis, culling, and strict biosecurity measures.

The ASFV is a structurally complex double-stranded DNA virus with an ortho-icosahedral morphology, which is the only member of the *Asfarviridae* family. The ASFV genome is 190–193 kb and encodes >150 open reading frames, with the central regions of the genes being highly conserved [6,7]. The ASFV *CP204L* gene encodes structural protein p30, which is located in the cytoplasm of host cells. The p30 protein is an early and abundantly expressed protein with a molecular mass of 34 kDa and good immunogenicity [8,9]. It is also one of the most antigenic proteins in the ASFV and is involved in viral internalization after adsorption to the host cell [9,10]. Therefore, p30 is an important target for early diagnosis of ASF. The ASFV *B646L* gene encodes the p72 major coat protein [11], which enables the classification of the ASFV into 23 different genotypes. Morphologically, the ASFV has an icosahedral shape with a complex structure consisting of multiple concentric layers [12]. The pB602L is a late-expressed nonstructural protein that acts as a molecular chaperone for the major structural protein p72, forming an abnormal “zipper-like” structure in the absence of pB602L rather than a hexahedral viral particle [13]. The pB602L is highly antigenic, which can be used to develop diagnostic tools for ASF [14,15]. Therefore, these two proteins may be important targets for detection.

**Figure 1:**
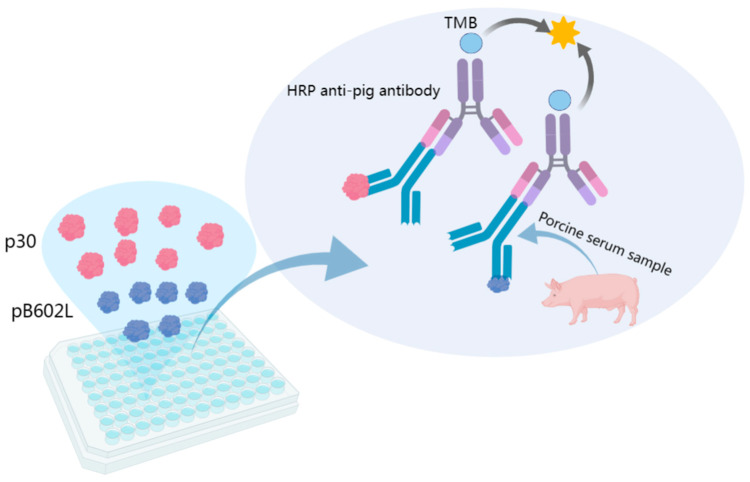
Schematic diagram of dual-antigen indirect ELISA principle. TMB: 3,3,5,5-tetramethylbenzidine.

As there is no effective vaccine or drug available for ASF control, early detection, diagnosis, and prevention are key in the prevention and control of ASF. To date, several conventional laboratory diagnostic techniques have been used for the detection of ASF, including polymerase chain reaction (PCR), loop-mediated isothermal amplification (LAMP), fluorescent quantitative PCR, colloidal gold rapid test strips, and ELISA [16,17]. In particular, ELISA is the most commonly used specific antibody assay and is a designated experiment specified by WOAH (World Organization for Animal Health) for international trade to detect specific antibodies to ASFV [18]. ELISAs include direct, indirect, competition, and sandwich ELISAs, which have the advantages of being efficient, rapid, and easy to perform. Nevertheless, the development of a rapid, sensitive, and stable ASFV antibody detection method is particularly important for the early diagnosis and control of ASF on swine farms. The dual-antigen indirect ELISA involved in this study used two antigens to detect the target antibody; the study design and schematic graph are presented in (Figure 1), which had higher sensitivity and specificity compared with other methods. We expressed and purified ASFV p30 and pB602L proteins and established a highly sensitive and specific dual-antigen indirect ELISA method for detecting ASFV antibodies and preventing the occurrence of ASF.

## 2. Materials and Methods

### 2.1. Serum Samples

ASFV-positive sera were obtained from the China Veterinary Drug Inspection Institute (Beijing, China). Standard positive sera for porcine circovirus type 2 (PCV2), classical swine fever virus (CSFV), porcine reproductive and respiratory syndrome virus (PRRSV), and pseudorabies virus (PRV) were obtained from China Veterinary Culture Collection Center (Beijing, China). ASFV-negative sera (normal porcine serum was used as a negative control) and clinical serum samples were originally purchased from the China Veterinary Drug Inspection Institute (Beijing, China) and then stored in our laboratory as biological material for specialized research.

### 2.2. Expression and Purification of p30 and pB602L

**Figure 2:**
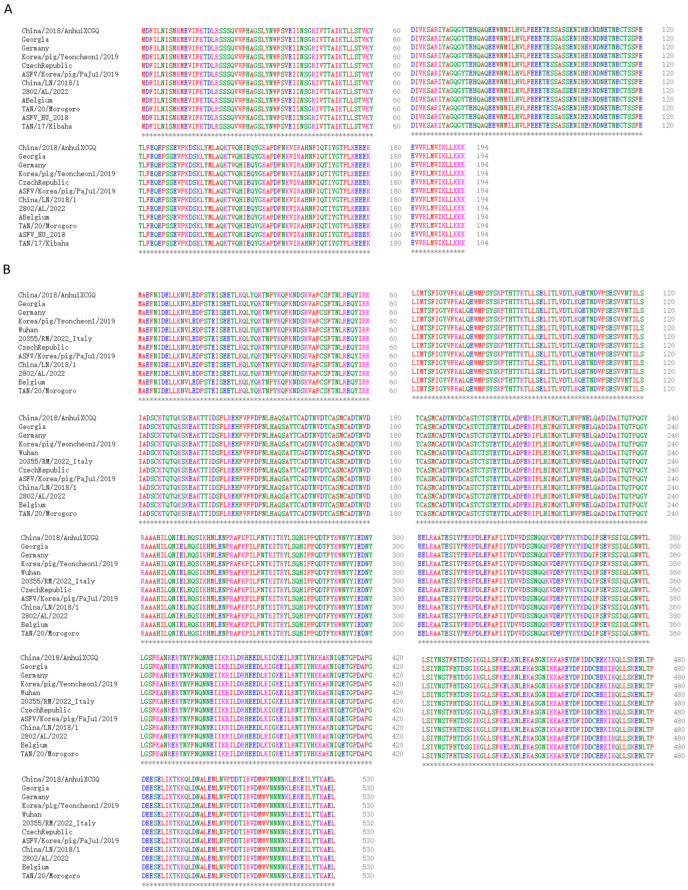
(**A**) Comparison of *CP204L* genes of different virulent strains. (**B**) Comparison of *B602L* genes of different virulent strains.

The full-length *CP204L* and *B602L* coding sequences were taken from the NCBI database (GenBank: MK128995.1) and synthesized by Tsingke Biotechnology Co., Ltd. (Zhengzhou, China). To construct expression plasmids for p30 and pB602L, p30 was amplified by PCR using primers containing *Bam*HI and *Eco*RI restriction sites for the *CP204L* sequence, while pB602L was amplified by PCR using primers for *Not*I and *Eco*RI restriction sites for the *B602L* sequence (Table 1). Multiple sequence comparisons assessed the stability of *CP204L* and *B602L* in different ASFV strains (Figure 2A,B). *CP204L* and *B602L* protein sequences were in perfect agreement with those in other major prevalent strains, including Georgia 2007/1, Korea/pig/Yeoncheon1/2019, Wuhan 2019-2, and 2802/AL/2022 Italy. Therefore, these two sequences were chosen to be sufficient for the detection of multiple strains prevalent in the country [19]. Following sequencing and identification, the *CP204L* and *B602L* sequences were homologously recombined into the pET-30a (+) and pET-28a (+) vectors. The obtained recombinant plasmids were transformed into *Escherichia coli* DH5α recipient cells, transferred into *E. coli* BL21 (DE3) recipient cells after sequence identification, and then optimized for incubation time, temperature, and isopropyl β-d-1-thiogalactopyranoside induction concentration. The supernatant was collected and centrifuged at 12,000 rpm for 1 h at 4 °C. The proteins were purified using a nickel-nitrilotriacetic acid metal affinity chromatography column, followed by SDS-PAGE and Coomassie brilliant blue staining for validation. The p30 and pB602L were analyzed using SDS-PAGE and Coomassie brilliant blue staining.

### 2.3. Western Blotting

Proteins were separated by SDS-PAGE and transferred to polyvinylidene difluoride membranes (Millipore, Billerica, MA, USA). Membranes were blocked with 5% skimmed milk in PBST (PBS with 0.05%, Tween-20) for 2 h. Membranes were incubated with standard ASFV-positive serum as primary antibodies (1:1000) at 4 °C overnight, followed by HRP-conjugated goat anti-mouse antibody (1:5000) for 40 min. Finally, the membranes were visualized and analyzed by the Amersham Imager 680 bioanalytical imaging system (Cytiva, Marlborough, MA, USA).

### 2.4. Establishment of Indirect ELISA Method Based on p30 and pB602L Dual-Antigen

The optimal antigen coating concentrations and serum dilutions were determined by checkerboard titration, and p30 and pB602L were diluted (1:100–1:1000) and coated on 96-well microtitration plates. ASFV-positive and ASFV-negative sera at different dilutions (1:10–1:800) were incubated. According to the molar ratio of the two proteins, p30and pB602L were encapsulated in a ratio of (5:1, 4:1, 3:1, 2:1, 1:1, 1:2, 1:3, 1:4, and 1:5), thus selecting the optimal encapsulation ratio. We used carbonate buffer (0.05 mol/L, pH 9.6), NaHCO_3_ (0.05 mol/L, pH 9.6) and phosphate buffer (PBS, pH 7.3) to encapsulate the antigen at 1 h at 37 °C, 2 h at 37 °C, 6 h at 4 °C, and overnight at 4 °C. The optimal closure solutions were selected from 3%, 5%, and 8% skimmed milk and 3%, 5%, and 8% bovine serum albumin and were closed at 37 °C for 1 h, 37 °C for 2 h, 4 °C for 6 h, and 4 °C overnight. The sera were incubated for 0.5, 1, 1.5, 2, and 2.5 h to select the optimal incubation time. The enzyme-labeled antibodies were incubated at the ratios of 1:2000, 1:4000, 1:6000, 1:8000, and 1:10,000, and the reaction times were 0.25, 0.5, 0.75, 1, and 1.5 h to obtain the optimal reaction conditions. After five rounds of washing, the chromogenic solution was added and incubated at ambient temperature and protected from light for 5, 10, 15, 20, and 25 min. Finally, the reaction was terminated by adding 50 μL 2 M sulfuric acid termination solution, and the absorbance was measured at 450 nm using an enzyme marker. The assay was repeated twice for all samples.

### 2.5. Determination of the Cutoff Value

Fifty negative clinical sera were used to calculate the thresholds for the dual-antigen indirect ELISA. The average ($\bar{X}$) and standard deviation (SD) of OD_450_ were calculated by statistical analysis. The cutoff value was determined as ($\bar{X}$ + 3SD). When OD_450_ of the sample was greater than or equal to the cutoff value, it was determined to be positive. If not, it was determined to be negative.

### 2.6. Sensitivity and Specificity Determination

To verify the specificity of the established dual-antigen indirect ELISA, CSFV, PRRSV, PCV2, PRV, ASFV-positive sera, and ASFV-negative sera were tested under optimized conditions. The sensitivity of the method was determined by diluting ASFV-positive sera from 1:10 to 1:6400 according to the optimized conditions, reading the OD_450_ value, and observing the change in this value with increasing serum dilution.

### 2.7. Reproducibility of Indirect ELISA with Dual-Antigen

To evaluate the reproducibility of the dual-antigen indirect ELISA, intra- and inter-batch reproducibility of the established indirect ELISA was determined using five positive and five negative sera from known backgrounds. For intra-batch reproducibility, each sample was repeated five times on ELISA plates coated at the same time. For inter-batch reproducibility, each sample was repeated five times on ELISA plates coated with different batches. Results are expressed as coefficient of variation (CV), which is the ratio of SD to mean OD_450_ value for each group of samples.

### 2.8. Comparison of Dual-Antigen Indirect ELISA with Commercial Kits

All clinical serum samples were compared and analyzed by the commercial ASFV-blocking ELISA antibody detection kit, the product which is mainly based on the detection of ASFV p30 antibody, (Qingdao Lijian Biotechnology Co., Ltd., Qingdao, China) in comparison with the established dual-antigen indirect ELISA method.

### 2.9. Statistical Analysis

All data were analyzed using GraphPad Prism 9.5.0 for raw letter analysis (GraphPad Software, San Diego, CA, USA).

## 3. Results

### 3.1. Expression and Purification of p30 and pB602L

**Figure 3:**
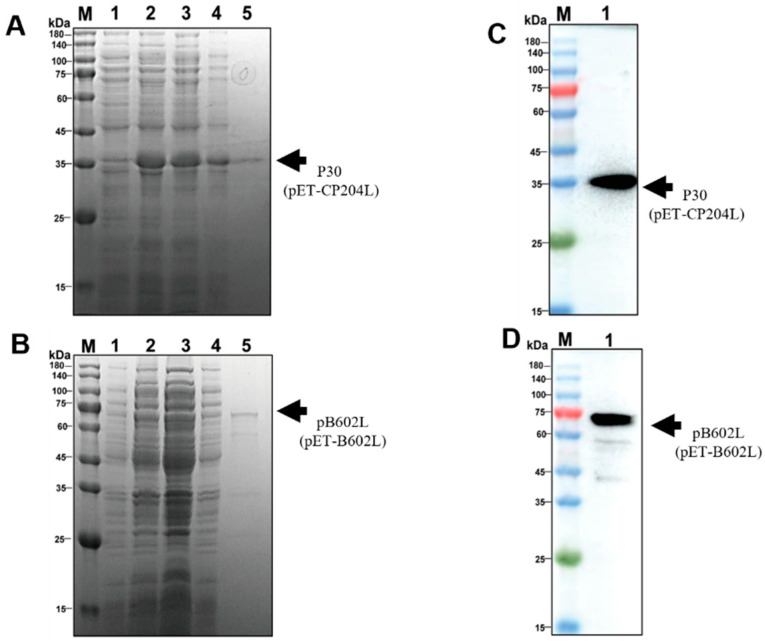
Solubility analysis and purification of p30 and pB602L proteins: (**A**,**B**) solubility analysis of p30 protein (**A**) and pB602L protein; (**B**) M: protein marker; (1): before induction; (2): after induction; (3): supernatant after sonication; (4): precipitation after sonication; (5): results of protein purification; (**C**,**D**) Western blot analysis of purified p30 and pB602L proteins. ASFV-positive serum was used as the primary antibody (dilution 1:1000); M: protein marker; (1): p30 protein (**C**) and pB602L protein (**D**).

To explore the optimal conditions for p30 and pB602L expression, recombinant *E. coli* containing pET-CP204L and pET-B602L was incubated at 37 °C, and when OD_600_ reached 0.5–0.8, 0.5 mM isopropyl β-d-1-thiogalactopyranoside was added to induce 12–14 h at 16 °C. After purification by nickel column, p30 was obtained at ~34 kDa and pB602L at ~68 kDa (Figure 3A,B). Western blotting showed that purified p30 and pB602L reacted specifically with ASFV-positive sera (Figure 3C,D). Purified p30 and pB602L had high immunogenicity.

### 3.2. Optimization of Optimal Conditions for Indirect ELISA with Dual-Antigen

By checkerboard titration, the OD values of positive (P) and negative (N) sera were maximum (P/N value of 24.732) when the dilutions of antigen and serum were 0.6 µg/mL and 1:200, respectively (Table 2). Therefore, the final concentration of the encapsulated antigen was calculated as 600 ng/well, and the optimal dilution of the serum was 1:200. Based on the molar ratio of the two proteins, the optimal encapsulation ratio of p30 and pB602L was 4:1 (Table 3). The reaction temperature, time, and other conditions were also optimized using the P/N value as an indicator.

**Figure 4:**
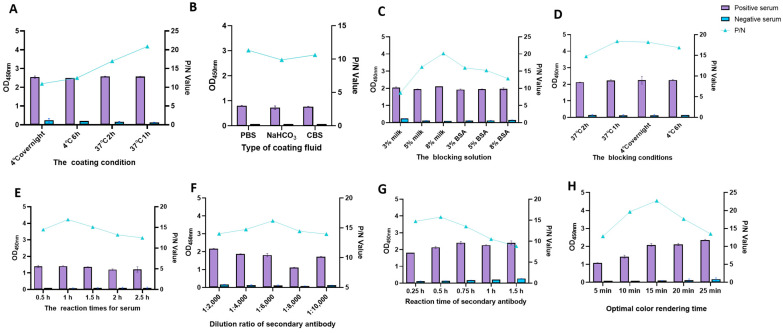
Optimization of dual-antigen indirect ELISA procedure: (**A**) determination of optimal coating conditions; (**B**) determination of the best coating solution; (**C**) determination of the best blocking solution; (**D**) determination of optimal blocking conditions; (**E**) optimal incubation time for serum; (**F**): dilution ratio of secondary antibody; (**G**) reaction time of secondary antibody; (**H**) optimal color rendering time.

The optimal coating conditions were screened from different coating temperatures and times, including 4 °C overnight, 4 °C for 6 h, 37 °C for 2 h, and 37 °C for 1 h. The optimal reaction condition was finally determined to be 37 °C for 1 h with maximum P/N (Figure 4A). The coating solutions included carbonate buffer (0.05 mol/L, pH 9.6), NaHCO_3_ (0.05 mol/L, pH 9.6), and PBS (pH 7.3), and PBS was selected as the optimal coating solution (Figure 4B). The optimal closure conditions, closure solution, serum reaction time, enzyme-labeled antibody dilution ratio, and reaction time were optimized sequentially under the determined optimal conditions. The optimal conditions for the dual-antigen indirect ELISA were to coat p30 and pB602L with PBS at a 4:1 ratio for 1 h at 37 °C, followed by 8% skimmed milk powder for 1 h at 37 °C. The optimal incubation time for serum was 1 h at 37 °C and 30 min at a dilution of 1:6000 for enzyme-labeled secondary antibody. The optimal color development time was 15 min at room temperature and protected from light (Figure 4).

### 3.3. Determination of Cutoff Values

**Figure 5:**
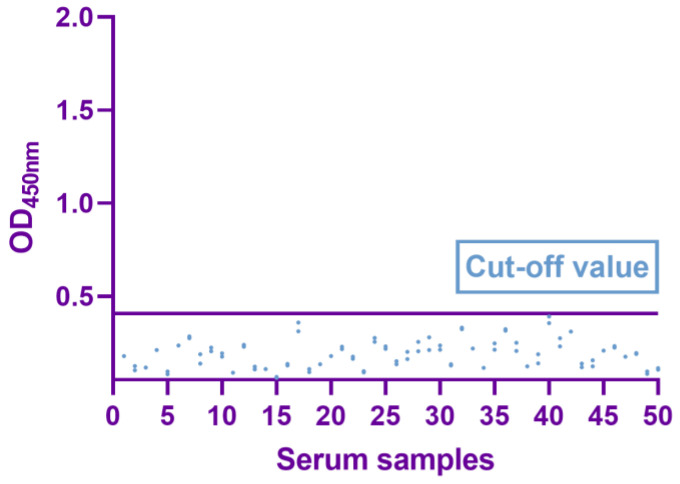
Determination of cutoff value of dual-antigen indirect ELISA (OD_450_ 0.407).

The optimal circumstances for testing 50 negative sera resulted in $\bar{X}$ of 0.184 and SD of 0.072, with a final critical value of $\bar{X}$ + 3SD = 0.403 (Figure 5). Serum specimens with OD_450_ greater than or equal to the critical value were deemed positive; if not, they were negative.

### 3.4. Sensitivity and Specificity Tests of Dual-Antigen Indirect ELISA

**Figure 6:**
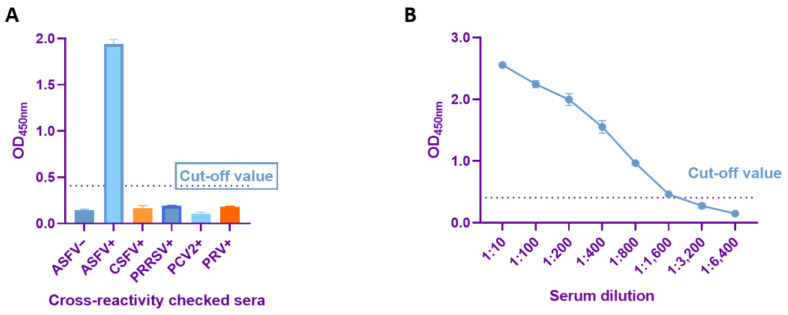
Sensitivity and specificity of dual-antigen indirect ELISA. (**A**) Specificity test of dual-antigen indirect ELISA. Dual-antigen indirect ELISA detected no cross-reactions with sera containing antibodies against four other porcine pathogens, including CSFV, PRRSV, PCV2, and PRV. (**B**) Determination of sensitivity.

The dual-antigen indirect ELISA with optimized conditions was used to detect PRRSV, PCV2, CSFV, PRV, ASFV-positive serum samples, and ASFV-negative serum samples, to evaluate the specificity of the method. The OD_450_ values of all serum samples were lower than the cutoff values, except for the ASFV-positive sera, whose OD_450_ values were higher than the cutoff values, indicating that the method had good specificity (Figure 6A). To evaluate the sensitivity of the method, ASFV-positive sera were diluted to 1:6400 and showed that the sensitivity of the dual-antigen indirect ELISA was 1:1600 (Figure 6B).

### 3.5. Repeatability Test

To evaluate the reproducibility of this ELISA, we determined five ASFV-positive serum samples by intra- and inter-batch reproducibility assays. The intra-batch CV ranged from 1.02% to 4.96% and the inter-batch CV from 3.04% to 8.98%, which suggested that the method had high reproducibility (Table 4).

### 3.6. Detection of Clinical Serum Samples

**Figure 7:**
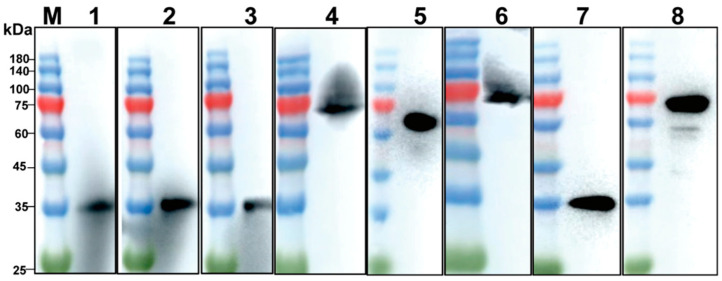
Western blotting of three clinical sera. M: protein marker. Results show lanes 1–6: three controversial clinical serum samples. Lanes 1–3: the p30 positive sera; lanes 4–6: the pB602L positive sera; lane 7: standard p30 positive serum control; lane 8: standard pB602L positive serum control.

We analyzed 106 serum samples using the established dual-antigen indirect ELISA and commercial kits. There were 103 samples in agreement with the commercial kits. Three sera tested positive with the dual-antigen indirect ELISA but negative with the commercial kits (Table 5). Western blotting was used to confirm the results, which showed that all three were positive (Figure 7), indicating that the dual-antigen indirect ELISA had higher accuracy.

## 4. Discussion

When the ASF pandemic was first reported in China in 2018, it quickly spread to almost all provinces and caused significant economic losses [20]. However, in the absence of an effective vaccine and drug treatment, highly sensitive and specific diagnostic methods have a critical role to play in the detection of ASF. Serological detection and molecular diagnostic methods are still considered the main tools for identifying and combating ASF [21,22], including ELISAs, immunoblots, indirect immunofluorescent antibody tests, lateral flow tests, and indirect immunoperoxidase tests; several of these tests are recommended by the WOAH for disease surveillance and for determining whether animals are infected with ASFV [4,23,24,25]. Although the molecular diagnostic method has high sensitivity, it also has stringent experimental and operational requirements. Therefore, ELISA is the most commonly used high-throughput serological detection method for ASF, with the advantages of low cost, high sensitivity, and strong specificity and it requires few special instruments and facilities to complete the detection. This means that ELISAs can facilitate large-scale batch detection on pig farms, so they are recommended as the main method for detecting ASFV antibodies [26,27].

Each ASFV-encoded protein is expressed in different phases, so it is essential to establish ELISA based on the proteins expressed in different phases of viral infection. Several commercial ELISA kits are available for the detection of ASFV antibodies (Ingenasa, Spain; ID. Vet, France; and Svanovir, Sweden). For example, the antigen used in the Ingenasa blocking ELISA kit is a purified p72 protein extract of the ASFV, which is the major structural protein (capsid protein) and the main antigen in infected pigs [28,29]. Because different ASFV proteins have different properties and exist in different phases, it is necessary to continuously explore the methods of ASFV-specific antibody detection, select different antigen combinations, and improve the ELISA. To improve the ELISA reactivity, we used the combined antigens p30 and pB602L because previous studies [30] showed that the combined application of two proteins (p30 and p54) improved sensitivity [31]. The p30 is an early expressed structural protein that is localized in the cytoplasm of infected cells and plays an important role in viral internalization [32]. The pB602L has promising antigenicity and immunogenicity. It can be used as a candidate antigen for ASF diagnostic methods, and a persistent high serological response to pB602L has been observed after infection in domestic and wild pigs infected with different viral isolates [14,33]. The pB602L contains a central variable region that allows frequent subgenotyping of ASFV isolates based on this region [34]. Therefore, in this study, the prokaryotic expression system was used to express ASFV p30 and pB602L. The two proteins obtained were soluble, which validated their antigenicity, and an ELISA for detecting ASFV antibodies was established on the basis of this method. We selected one early-stage and one late-stage antigen to detect the target antibody, which improved the sensitivity and specificity of the test and enabled the detection of different periods of ASF infection, thus detecting infection more accurately.

In summary, the established dual-antigen indirect ELISA method did not cross-react with antibodies of other swine viruses, such as PRRSV, CSFV, PCV2, and PRV. The maximum dilution of the sera could detect ASFV-positive sera at 1:1600, indicating good sensitivity of the method. The intra-batch reproducibility of the assay was CV < 5% and the inter-batch reproducibility was CV < 10%, indicating good reproducibility. Compared with commercial kits, the dual-antigen indirect ELISA had good detection performance. The current study provided a new platform for ASFV antibody detection, although the method still needs further validation with large samples.

## Figures and Tables

**Table 1:** Primer sequences.

Primers Name	Primers Sequences
CP204L-F	AGCTTGTCGACGGAGCTCGAATTCTTATTTTTTTTTTAA
CP204L-R	CAAGGCCATGGCTGATATCGGATCCATGGATTTTATTTTA
B602L-F	TGGTGGTGCTCGAGTGCGGCCGCCAATTCTGCTTT
B602L-R	ATGGGTCGCGGATCCGAATTCATGGCAGAATTTAA

**Table 2:** Determination of optimal antigen coating concentration and serum dilutions.

Dilution of Sera		Antigen at Different Concentrations (µg/mL)					
		0.1	0.2	0.4	0.6	0.8	1
1:10	P	0.847	1.273	1.691	1.961	2.168	2.105
	N	0.159	0.180	0.362	0.249	0.236	0.251
	P/N	5.333	7.084	4.669	7.876	9.208	8.394
1:50	P	0.503	0.813	1.413	1.840	1.905	2.031
	N	0.103	0.084	0.086	0.104	0.114	0.140
	P/N	4.871	9.729	16.527	17.680	16.686	14.562
1:100	P	0.381	0.691	1.337	1.724	1.780	1.851
	N	0.060	0.171	0.082	0.076	0.081	0.096
	P/N	6.304	4.038	16.263	22.571	22.100	19.376
1:200	P	0.308	0.603	1.013	1.621	1.546	1.567
	N	0.057	0.063	0.057	0.066	0.071	0.072
	P/N	5.385	9.579	17.744	24.732	21.935	21.729
1:400	P	0.226	0.349	0.616	1.141	1.094	1.096
	N	0.066	0.094	0.056	0.084	0.066	0.084
	P/N	3.406	3.697	11.012	13.523	16.683	12.991
1:600	P	0.179	0.313	0.528	0.940	0.984	1.067
	N	0.056	0.064	0.058	0.057	0.060	0.068
	P/N	3.219	4.900	9.038	16.447	16.388	15.721
1:800	P	0.175	0.305	0.443	0.950	0.921	0.952
	N	0.056	0.058	0.064	0.062	0.060	0.061
	P/N	3.113	5.287	6.972	15.277	15.284	15.518

Notes. The black bold value indicates the value under the optimal condition chosen for subsequent indirect ELISA with dual antigen. P: OD value of positive samples; N: OD value of negative samples.

**Table 3:** Coating volume ratio of p30 and pB602L.

Volume Ratio of p30 to pB602L	5:1	4:1	3:1	2:1	1:1	1:2	1:3	1:4	1:5
P	1.061	1.024	0.914	0.834	0.675	0.500	0.502	0.424	0.367
N	0.090	0.056	0.059	0.055	0.084	0.057	0.080	0.066	0.063
P/N	11.783	18.204	15.564	15.231	8.045	8.713	6.274	6.442	5.800

Notes. The black bold value indicates the value under the optimal condition chosen for subsequent indirect ELISA with dual antigen. P: OD value of positive samples; N: OD value of negative samples.

**Table 4:** Results of the repeatability assay for dual-antigen indirect ELISA.

Sample No.		Intra-Assay CV (%)		Inter-Assay CV (%)	
		X ± SD	CV (%)	X ± SD	CV (%)
Positive samples	1	1.689 ± 0.070	4.14	1.492 ± 0.064	4.32
	2	1.842 ± 0.019	1.02	1.424 ± 0.043	3.04
	3	1.571 ± 0.057	3.65	1.455 ± 0.069	4.71
	4	1.918 ± 0.047	2.44	1.493 ± 0.063	4.21
	5	1.962 ± 0.040	2.04	1.458 ± 0.062	4.24
Negative samples	6	0.107 ± 0.005	4.96	0.110 ± 0.006	5.87
	7	0.073 ± 0.003	4.39	0.068 ± 0.003	5.11
	8	0.085 ± 0.003	3.95	0.091 ± 0.006	6.98
	9	0.087 ± 0.002	2.28	0.070 ± 0.006	8.98
	10	0.083 ± 0.004	4.44	0.071 ± 0.005	7.05

**Table 5:** Comparison of dual-antigen indirect ELISA and commercial kits.

No. of Clinical Samples	Dual-Antigen Indirect ELISA		Commercial Kits	
	No. of Positive	Positive Rate (%)	No. of Positive	Positive Rate (%)
106	17	16.1%	14	13.3%

## Data Availability

The data that support the findings of this study are available from the corresponding author upon reasonable request.
